# Supplementary material for: Pitfalls in Single Clone CRISPR-Cas9 Mutagenesis to Fine-Map Regulatory Intervals
Source: Genes (Basel). 2020 May 4;11(5):504. doi: 10.3390/genes11050504 (PMC7288657; doi:10.3390/genes11050504)
Supplement: Supplementary file 1 [file genes-11-00504-s001.zip › Supp Figures.pdf]

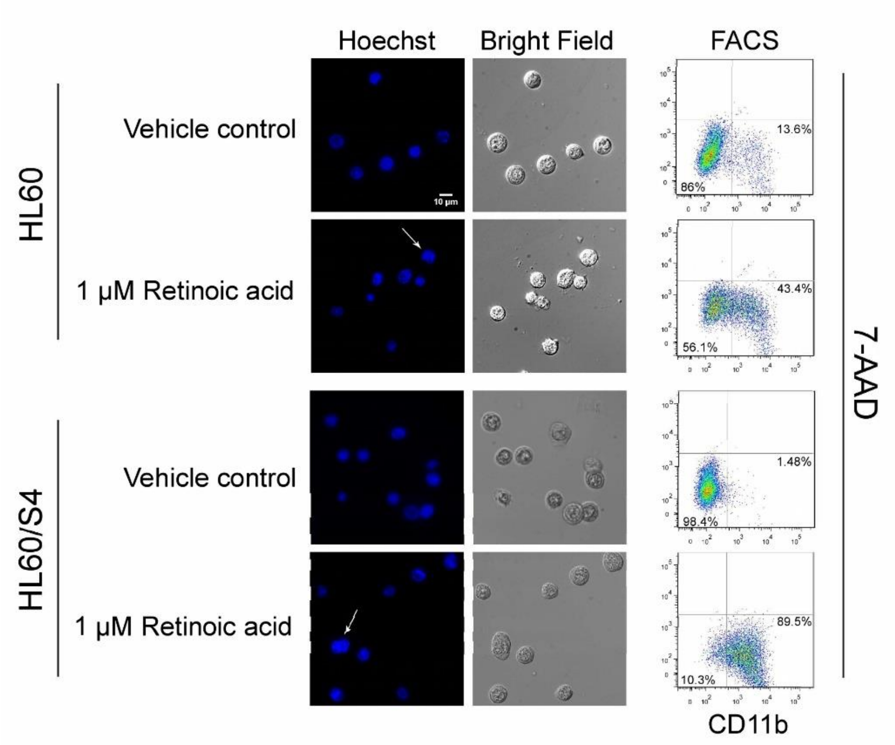

Supplementary Figure 1. Characteristics of neutrophils by immunofluorescence and flow cytometry analysis. Left two panels show the Hoechst staining of multilobular nuclei of differentiated neutrophils. A representative figure of one clone of each cell type, HL60 and HL60/S4. The cell pointed by the arrow represents two lobes of the nucleus. The right panel shows flow cytometry analysis of CD11b (cell surface marker for neutrophil) expression and 7-AAD staining to stain dead cells. The gated populations with cell proportion show CD11b-/7-AAD+, live undifferentiated cells (bottom left quadrant) and CD11b+/7-AAD-, live differentiated cells (bottom right quadrant).

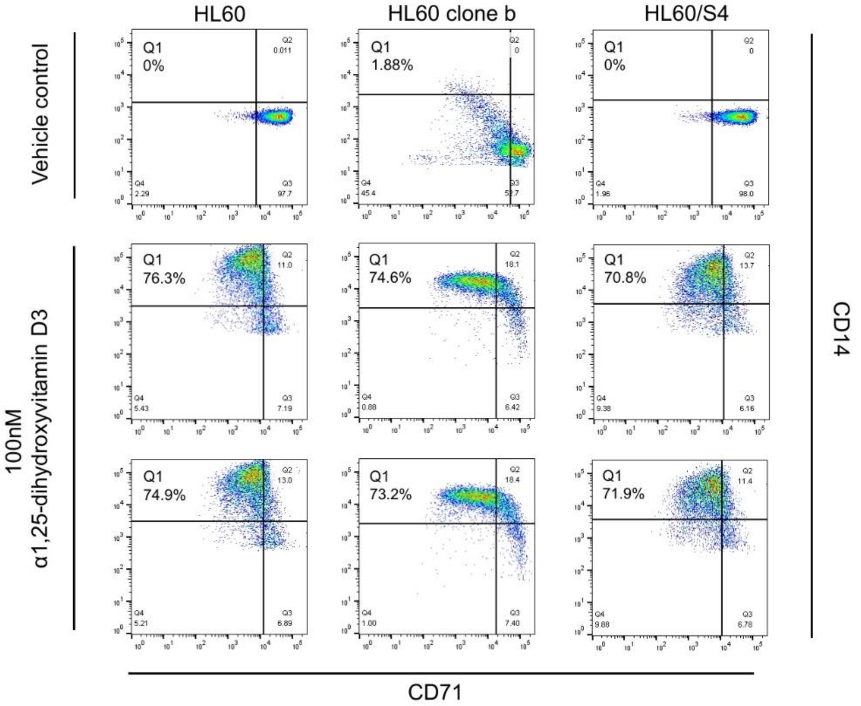

Supplementary Figure 2. Characteristics of monocytes by flow cytometry analysis. The flow cytometry analysis of CD71 and CD14 expression in control and monocyte differentiated HL60 bulk, HL60 clone b and HL60/S4 bulk cells. The gated population (Q1) with cell proportion shows monocytes (CD71-/CD14+).

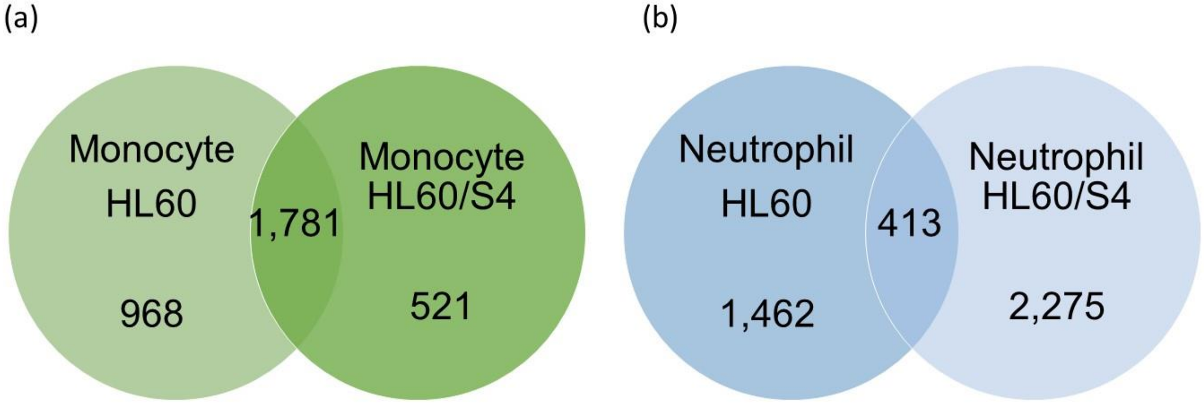

Supplementary Figure 3. Venn diagram of differential expressed genes. The Venn diagrams show the number of significant inter-clonal differentially expressed genes (FDR<0.0001) in monocyte lineage (a) and neutrophil lineage (b) differentiation in HL60 and HL60/S4.

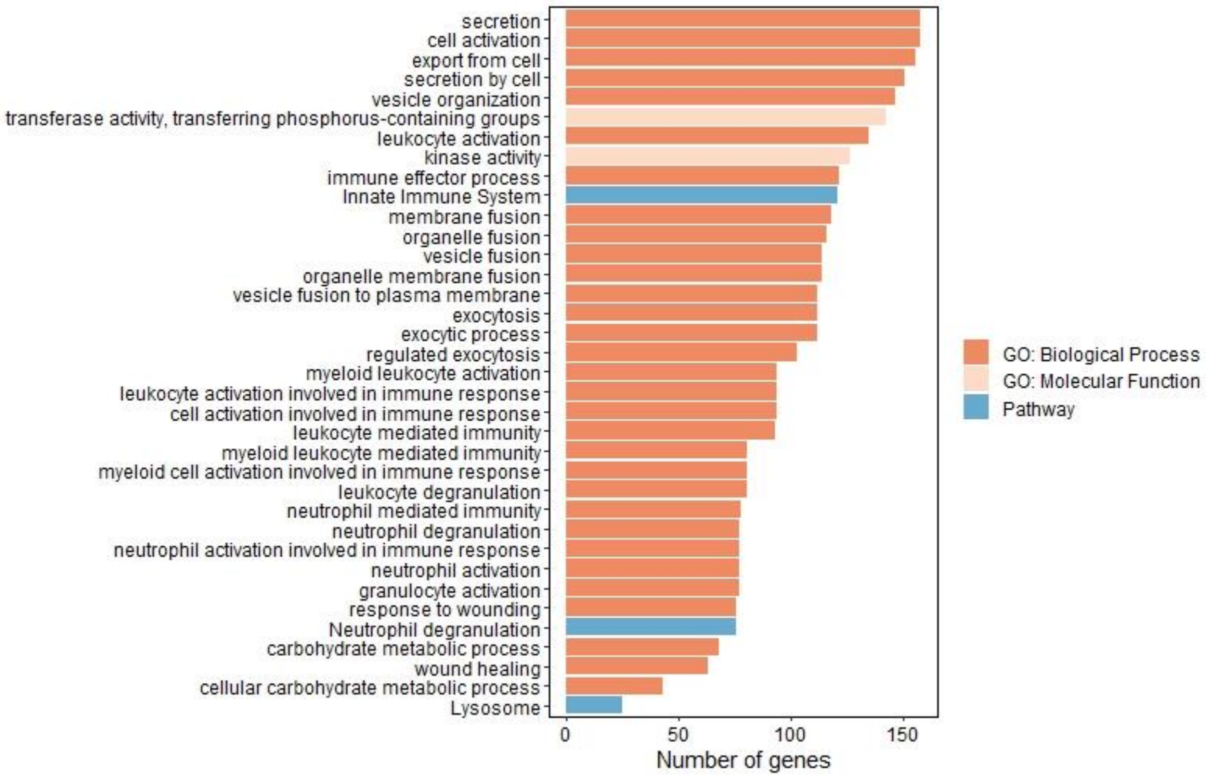

Supplementary Figure 4. Gene ontology and pathway analysis of differential expression genes in HL60 monocyte derivatives. Uniquely differentially expressed genes in HL60 monocytes (968) were used as input to perform gene ontology and pathway analysis by Toppfun. Gene ontology terms involving in biological process and molecular function, and pathways are categorized and listed on the vertical x-axis. And the number of DE genes involved in each term is shown on the horizontal y-axis. P-value is corrected by Bonferroni correction with 0.00001 as cut-off.

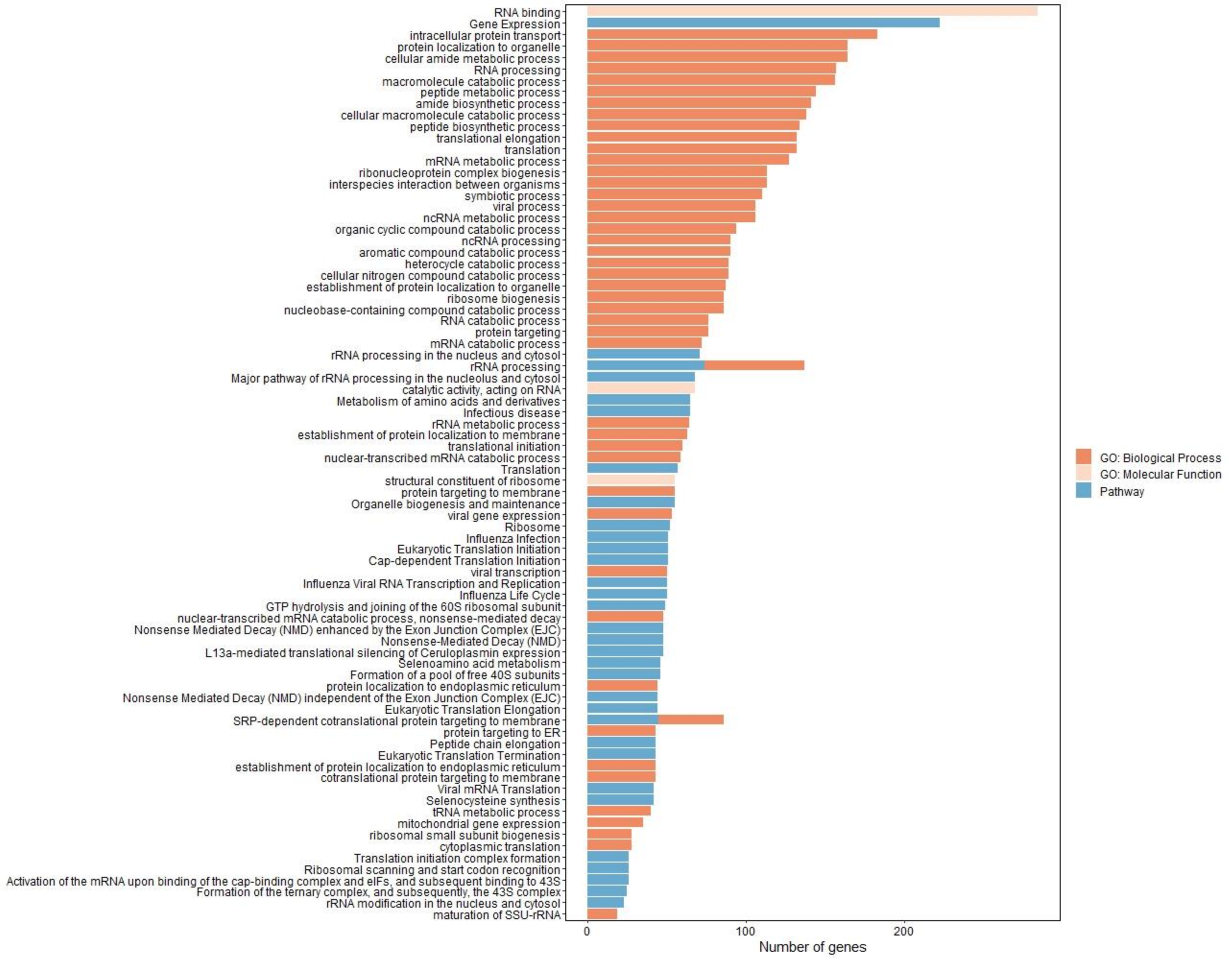

Supplementary Figure 5. Gene ontology and pathway analysis of differential expression genes in HL60 neutrophil derivatives. Uniquely differentially expressed genes in HL60 neutrophil (1,462) were used as input to perform gene ontology and pathway analysis by Toppfun. Gene ontology terms involving in biological process and molecular function, and pathways are categorized and listed on the vertical x-axis. And the number of DE genes involved in each term is shown on the horizontal y-axis. P-value is corrected by Bonferroni correction with 0.00001 as cut-off.

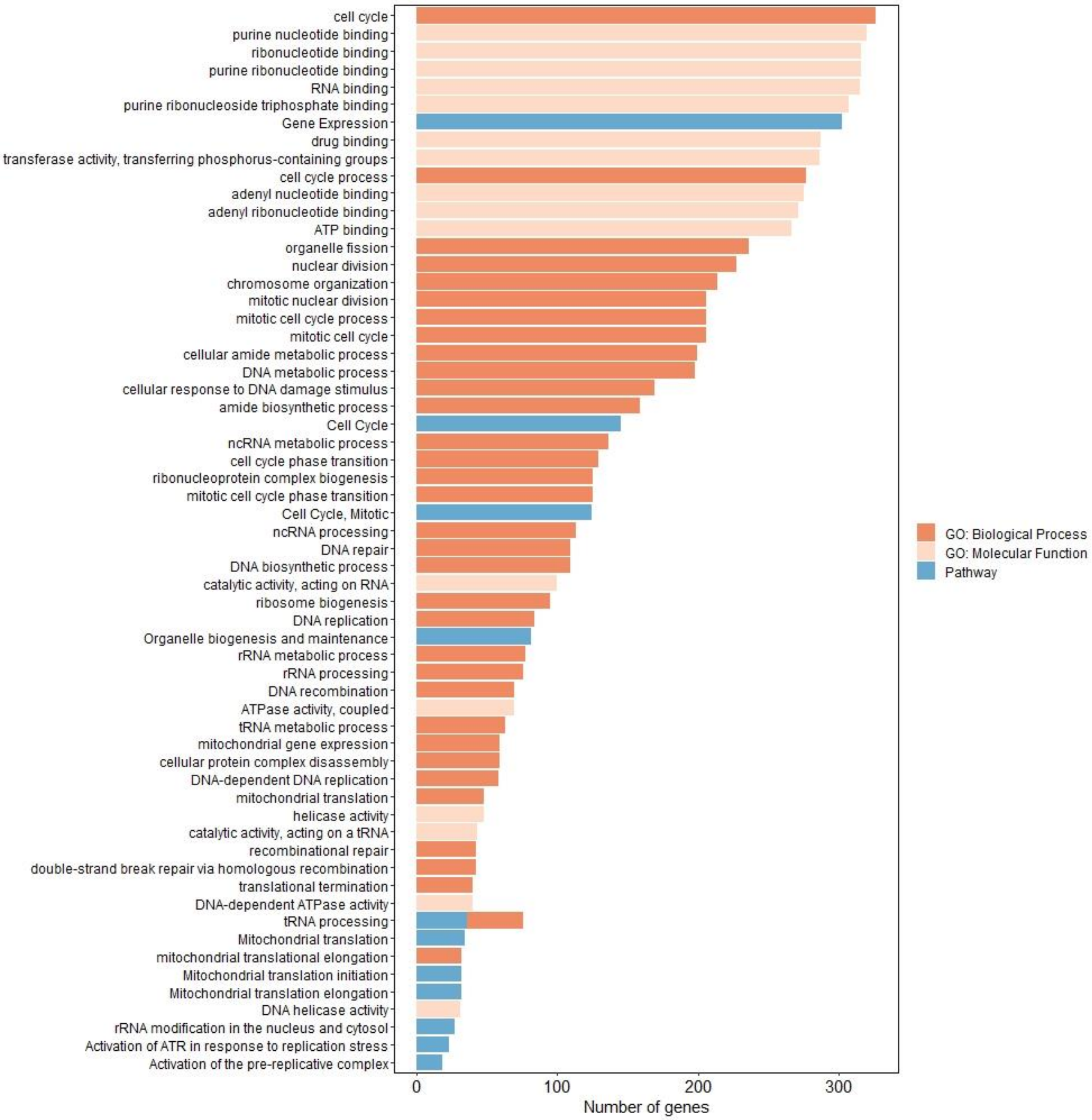

Supplementary Figure 6. Gene ontology and pathway analysis of differential expression genes in HL60/S neutrophil derivatives. Uniquely differentially expressed genes in HL60/S4 neutrophil (2,275) were used as input to perform gene ontology and pathway analysis by Toppfun. Gene ontology terms involving in biological process and molecular function, and pathways are categorized and listed on the vertical x-axis. And the number of DE genes involved in each term is shown on the horizontal y-axis. P-value is corrected by Bonferroni correction with 0.00001 as cut-off.

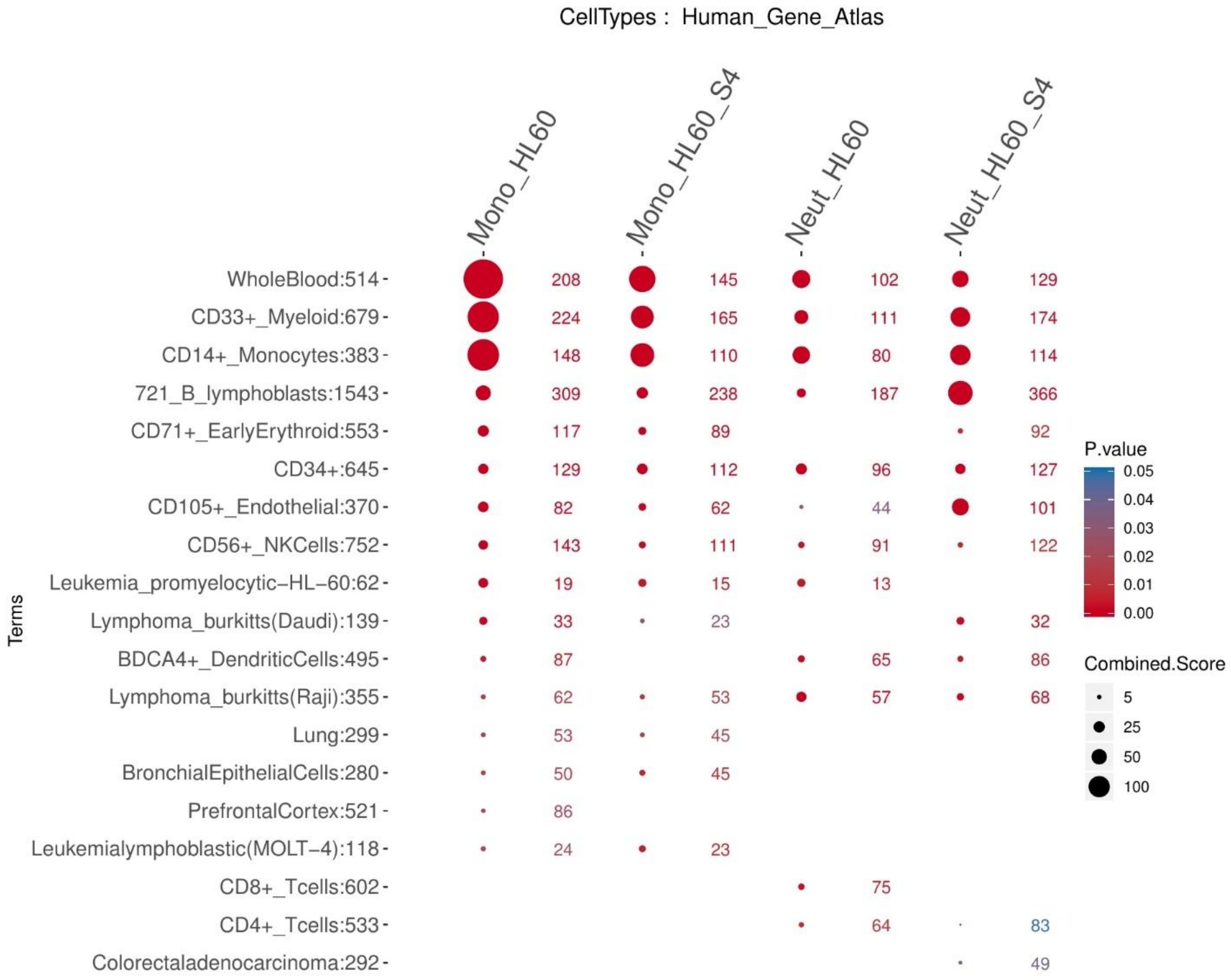

Supplementary Figure 7. Gene ontology enrichment analysis of differential expression genes in HL60 and HL60/S4 monocyte and neutrophil derivatives. Uniquely differentially expressed genes in HL60 monocytes (968), HL60/S4 monocytes (521), HL60 neutrophils (1,462), HL60/S4 neutrophils (2,275) were used as input to perform gene ontology enrichment analysis by Enrichr. The total number of genes in each cell type term is shown on the y-axis and the number of DE genes enriched in each term is shown next to each dot. P-value and combined score (log(p-value) × z-score) are coded by the color and size of dots, respectively.

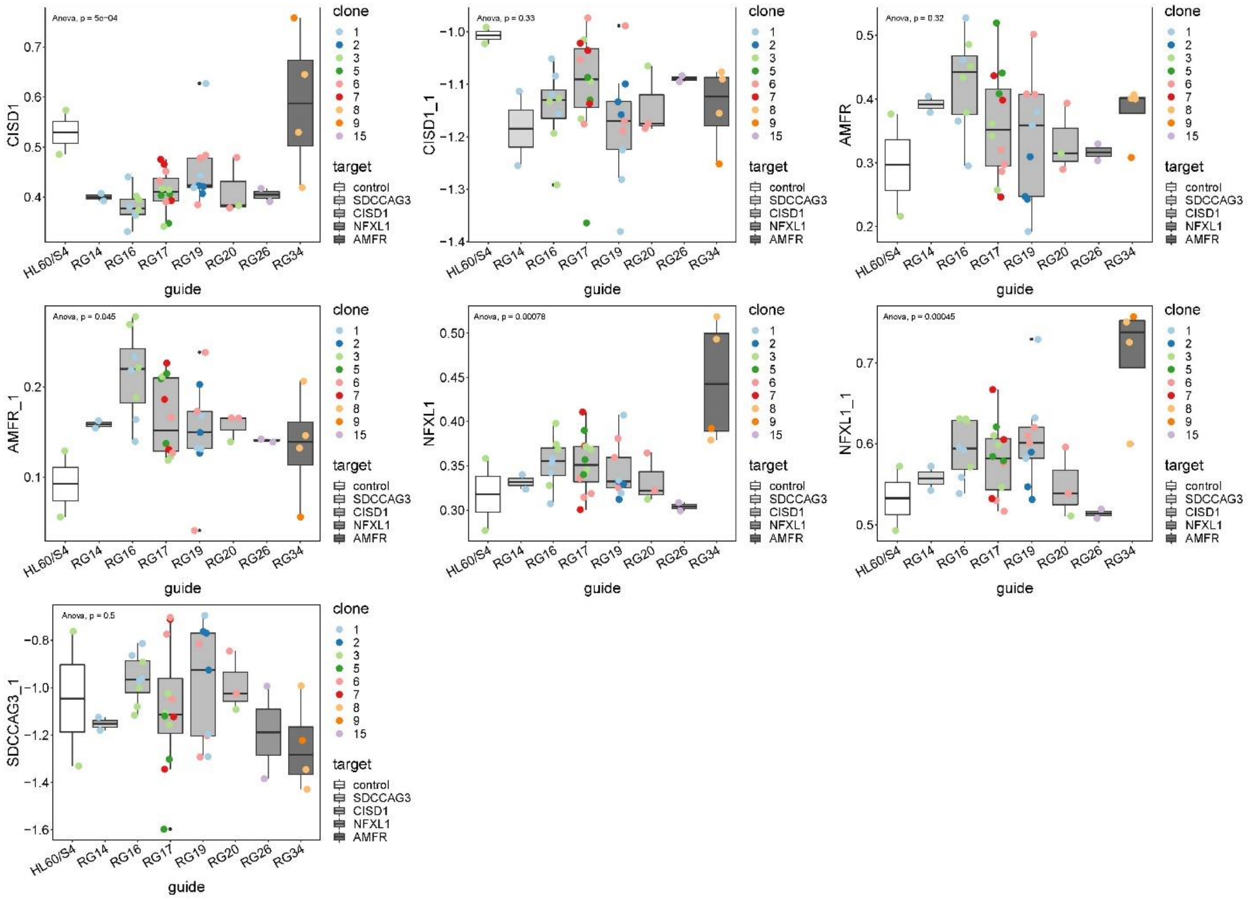

Supplementary Figure 8. Quantification of all targeted gene expression normalized by GAPDH in all CRISPR-Cas9 edited single cell clones by Fluidigm qRT-PCR. Single cell clones were grouped by guide RNA and the expression of seven probes was shown as boxplot across all clones within each guide RNA group. Clones with the same genotype in each guide RNA group were colored coded. The value was normalized by the mean expression value of two probes of housekeeping gene, GAPDH. ANOVA test was performed to compare the mean differences in multiple groups, and the p-value was reported in each panel.
